# Effect of Annealing on Microstructures and Hardening of Helium-Hydrogen-Implanted Sequentially Vanadium Alloys

**DOI:** 10.1186/s11671-018-2485-6

**Published:** 2018-03-02

**Authors:** Shaoning Jiang, Zhiming Wang

**Affiliations:** School of Mechanical and Automotive Engineering, Qilu University of Technology (Shandong Academy of Science), Jinan, 250353 China

**Keywords:** Bubbles, Dislocation loops, Helium/hydrogen synergistic effect, Irradiation hardening

## Abstract

The effect of post-irradiation annealing on the microstructures and mechanical properties of V-4Cr-4Ti alloys was studied. Helium-hydrogen-irradiated sequentially V-4Cr-4Ti alloys at room temperature (RT) were undergone post-irradiation annealing at 450 °C over periods of up to 30 h. These samples were carried out by high-resolution transmission electron microscopy (HRTEM) observation and nanoindentation test. With the holding time, large amounts of point defects produced during irradiation at RT accumulated into large dislocation loops and then dislocation nets which promoted the irradiation hardening. Meanwhile, bubbles appeared. As annealing time extended, these bubbles grew up and merged, and finally broke up. In the process, the size of bubbles increased and the number density decreased. Microstructural changes due to post-irradiation annealing corresponded to the change of hardening. Dislocations and bubbles are co-contributed to irradiation hardening. With the holding time up to 30 h, the recovery of hardening is not obvious. The phenomenon was discussed by dispersed barrier hardening model and Friedel-Kroupa-Hirsch relationship.

## Background

Vanadium-based alloys have been developed for possible use in the structure of fusion power reactors because of their potential for low activation and their attractive high-temperature properties [1]. However, hydrogen (H) and helium (He) produced by nuclear transmutation reaction in fusion reactor would influence the microstructure and mechanical properties greatly [2]. On the basis of atom, He with low solubility affects greatly. He could promote irradiation hardening/embrittlement as well as segregation and void swelling [3, 4]. In addition, the potential synergistic effect of helium and hydrogen need further study during irradiation [5]. Research on irradiation hardening of V-4Ti after He+H irradiation indicated that He bubbles could not form in V-4Ti when the He concentration was less than 0.5 at.%. Therefore, the irradiation hardening for V-4Ti with H and He could mainly be defects formed during irradiation [6]. It is necessary to study the effect of high He and H concentrations on microstructures and hardening, in other words, how responsible the dislocation loops/nets and bubbles for the irradiation hardening. Kong et al. [7] studied the influence of Au ion irradiation damage on helium-implanted tungsten, used Orowan stress formula [8] to interpret the interaction between helium bubbles and irradiation defects in tungsten materials and found helium bubbles as impenetrable obstacles for the dislocation motion, and thought the density and size of helium bubbles were the key factors for hardening. Irradiation defects would also produce during irradiation. The relationship among defects, dislocation loops and bubbles needs further consideration.

Post-irradiation annealing was discussed for the recovery of irradiation damage and mechanical properties recently [9–11]. For post-irradiation annealing above 600 °C, the recovery of damage structure and tensile properties occurred and irradiation hardening completely disappeared in V-3Fe-4Ti-0.1Si. No significant recovery of irradiation hardening could be observed in the irradiated specimens after post-irradiation annealing at 500 °C for 2 h [12]. The research of irradiation damage of recovery by post-irradiation annealing of EUROFER base steels showed that repeated intermediate annealing treatment at 550 °C made RAFM steel withstood much higher nominal damage dose rates. After annealing, the embrittlement further decreased, while the hardening also decreased. Meanwhile, annealing at 500 °C was supposed to be the minimum temperature for starting recovery [13] of EUROFER base steels. Temperature below 500 °C should also be explored for the possibility of the recovery process from irradiation hardening in an operating mode maintenance process of a fusion reactor because the temperature will be kept in the regime where liquid lithium will circulate in the blanket module for cooling the decay heat after neutron exposure even in the suspension period of the operation of a fusion reactor. Investigating the recovery process from irradiation hardening and the post-irradiation annealing at a lower temperature would require a long-term annealing treatment to extend the temperature regime to a lower temperature and thus promote easier self-healing treatment in the reactor [14].

This study conducted experiments to determine the post-irradiation annealing effect on the microstructures and mechanical properties of He and H-irradiated V-4Cr-4Ti alloys. Four groups of samples (i.e., as-irradiated specimens and specimens that underwent the post-irradiation annealing treatment at 450 °C for 10, 20, and 30 h) were carried out by high-resolution transmission electron microscopy (HRTEM) observation and nanoindentation test. It aims to understand the thermal stability of the defect clusters and bubbles and investigate the recovery method for irradiation hardening.

## Methods/Experimental

V-4Cr-4Ti alloys were SWIP 30 from Southwestern Institute of Physics. Its chemical composition of main elements was as follows (Table 1).

**Table 1:** Chemical composition of main elements and impurities in V-4Cr-4Ti alloy (wt%)

Cr	Ti	C	N	O	S
3.81	3.92	0.013	0.0020	0.027	0.0020

V-4Cr-4Ti alloys were wrapped by Zr and Ta foils and sealed in high vacuum quartz capsules filled with pure argon then annealed at 1100 °C for 2 h. The annealed specimens were punched into discs with the gauge dimension of 100 μm thick and 3 mm diameter. Then, some of them were prepared to transmission electron microscope (TEM) samples after electropolishing. Others were polished for nanoindentation test. Both of them were irradiated He ions at first and then H ions at RT in ion accelerator from Beijing Radiation Center. Among which, the ion energy was 50 keV for He and 30 keV for H calculated by Stopping and Range of Ions in Matter (SRIM), which was chosen so that both ions had similar depth profiles. The irradiation dose for He and H ions was approximately 5 × 10^16^ ions/cm^2^, respectively. Post-irradiation annealing was carried out for 10–30 h at 450 °C with the same high vacuum condition as heat treatment. Microstructural observations were performed with FEI F-20 HRTEM. Nanoindentation test was conducted with Nano Indenter XP at RT. The indentation depth was 1000 nm, and nine indentations were chosen for each test.

## Results and Discussion

### Microstructural Observation

**Fig. 1:**
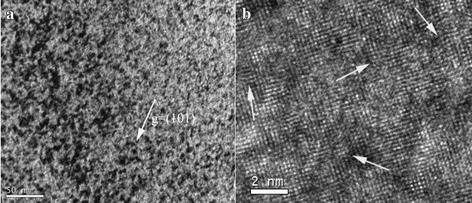
Images of V-4Cr-4Ti alloys after He+H ion irradiation sequentially at RT. **a** TEM bright-field image of defects. **b** HRTEM image of defects

TEM bright-field and HRTEM images of the irradiated V-4Cr-4Ti alloys are shown in Fig. 1. After He and H ion irradiation at RT, large amounts of defects appeared as shown in Fig. 1a. These defects included clusters of vacancies and interstitial atoms. Generally, both of them are produced with the same amount during irradiation. These defects distributed uniformly and were not distinguished one by one for the small dimension. Figure 1b shows the high-resolution image for V-4Cr-4Ti alloys after He and H irradiation at RT. There were some bending or break up of lattice fringe (white arrow). This is because fringe contrast is sensitive to defects. Therefore, the lattice fringe image showed abnormality during irradiation.

From Fig. 1, there was no visible bubble in irradiated He and H ions at RT. The nucleation of bubbles depends mainly on helium diffusion and temperature. Helium diffusion is a basic requirement for bubble nucleation and growth [3]. In irradiated He ions, He-vacancy (He-V) complexes were formed due to the high binding energy between He atom and vacancy [15], and a small number of He clusters. However, the mobility of He-V complexes and He clusters was limited or even negligible at RT which caused the suppression of bubble nucleation. Hydrogen ions continued to produce vacancies and interstitials. According to simulation, the binding of helium to clusters is always much stronger than that of hydrogen [16]. As a result, the newly produced vacancies induced by H irradiation were trapped by He-V complexes or He clusters. Hydrogen may be trapped by He-V clusters, or He clusters or very small helium bubble seed to help bubble nucleation [17].

**Fig. 2:**
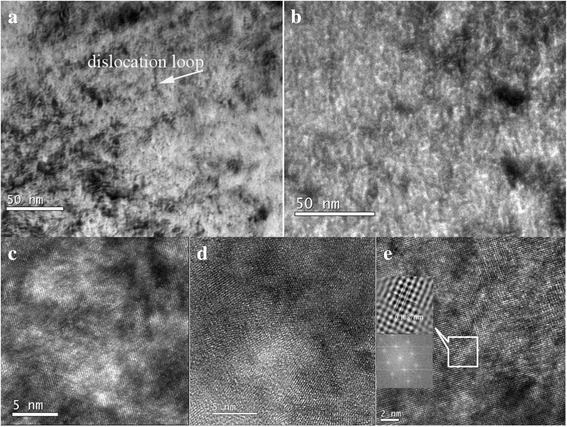
Dislocation loops and bubbles of V-4Cr-4Ti alloys after post-irradiation annealing treatment at 450 °C for 10 h. **a** Dislocation loops. **b** Bubbles of bright field. **c**, **d**, **e** Bubbles of high-resolution images

Figure 2 shows the images of He and H ion-implanted V-4Cr-4Ti alloy after annealing at 450 °C for 10 h. Figure 2a shows dislocation loops under focus, while Fig. 2b shows large amount of bubbles over focus. In situ TEM He^+^ implantation and annealing on nanocrystalline iron at RT also found two type of visible radiation damage: interstitial clusters and bubbles [18]. Both of them would increase the irradiation hardening of materials. Small dislocation loops which size is 4 nm were also observed in Fig. 2. The size and number density of the bubble are approximately 9 nm and 1.5 × 10^11^ cm^−2^, respectively. If implanted H ions are solely to tungsten at high temperature, small size hydrogen bubbles emerged. But bubbles in this study are supposed to be helium bubbles with some hydrogen. He atoms occupied vacancies, and hydrogen are trapped by He-V complexes, so the presence of He suppresses the formation of hydrogen bubbles [19].

After irradiation, the He and H contents are constant. With the increasing temperature, the mobility of He-V complexes increased and induced the formation of bubbles. Virtually, the nucleation of bubble occurs by the concurrent diffusion and clustering of He atoms, H atoms, vacancies (and interstitials) which represents a complicated nucleation process. However, the microstructures of irradiated annealed samples are dominated by not only bubbles but also dislocation loops/nets [20]. The nature of dislocation loops may be interstitial or vacancy type. Light ion irradiation such as helium and hydrogen at lower temperature resulted in interstitial loops [21]. Free interstitials migrate faster than vacancies which are involved in the strong formation of dislocation loops. So, in this study, the type of dislocation loops is interstitial.

**Fig. 3:**
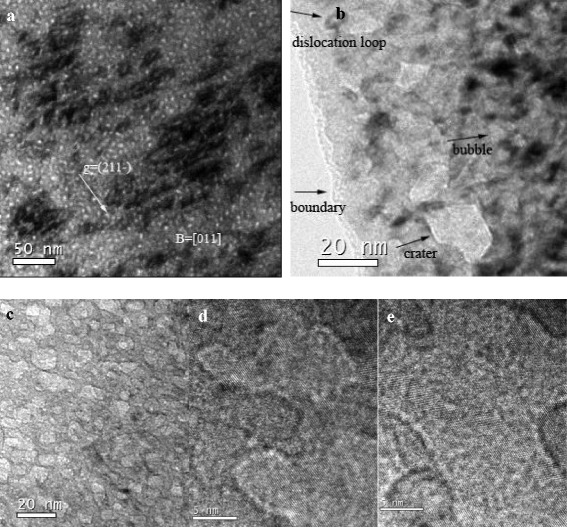
Microstructures of V-4Cr-4Ti alloys after post-irradiation annealing treatment at 450 °C for 20 h. **a** Dislocation loops of bright field. **b**, **c** Bubbles of bright field. **d**, **e** Bubbles of high-resolution images

With the increasing temperature or holding time, dislocation loops and bubbles that grew up and tended to coarsen are shown in Fig. 3, meaning that the average size increased while the density decreased with time. The microstructures were coexisting of large interstitial-type dislocation loops and bubbles. The mean size and number density of dislocation loops are 18 nm and 7.5 × 10^10^ cm^−2^, respectively. The mean size and number density of bubbles are 11 nm and 2.1 × 10^11^ cm^−2^.

During the continuing holding time, more and more He, H, vacancies, and small interstitials passed into the bubbles. The bubbles had higher pressure and larger volume. Finally, the over-pressurized bubbles which were close to the boundary of thin area ruptured firstly and flaked into crater (Fig. 3b) [22]. Meanwhile, vacancy and interstitial annihilate by all kinds of sinks like bubbles, loops, grain boundary, and surface.

The bubble coarsening is explained by Ostwald ripening mechanisms which is due to thermally activated resolution from small and re-absorption of He and H atoms by large bubbles [10, 23]. In addition, the pressure increased due to more and more He and H that enter into bubbles. Most hydrogen atoms were trapped by helium bubbles. During the process, hydrogen was supposed to be first attracted to the stress field of the highly pressurized helium bubbles. The coarsening of bubbles provides more free surface area to bind more hydrogen atoms.

**Fig. 4:**
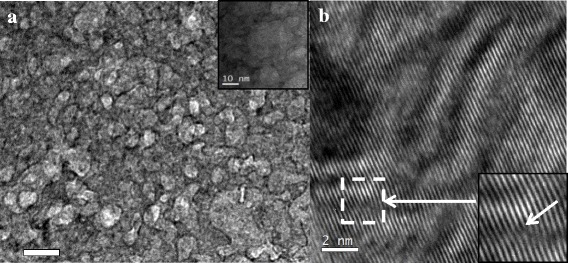
Microstructures of V-4Cr-4Ti alloys after post-irradiation annealing treatment at 450 °C for 30 h. **a** Bubbles of bright field. **b** Dislocation lines of high-resolution images

When the holding time was up to 30 h, the bubbles continued coarsening which are illustrated in Fig. 4. The mean size is 14 nm, and the number density is 1.6 × 10^11^ cm^−2^. The dislocation loops did not appear. A second mechanism that contributes to the decrease in dislocation loop density during annealing is the apparent escape of mobile loops at the free surface. This disappearance may result from either the rapid dissolution of point defects into the matrix or, more likely, the migration of the loop to the nearest sink, which in this case is the free surface [21]. From HRTEM image in Fig. 4b, we can identify dislocation lines.

Research about annealing above 400 °C in vanadium-based alloy found some Ti-O type plate-like and cuboidal precipitates [24]. To analyze the compositions of V-4Cr-4Ti alloy after post-irradiation annealing treatment (the holding time is 30 h), we used FEI Tecnai F20 microscope equipped with an energy dispersive X-ray spectrum (EDS) analysis system and scanning electron microscope (STEM-EDS) that carried out the composition analysis. The result is as follows.

**Fig. 5:**
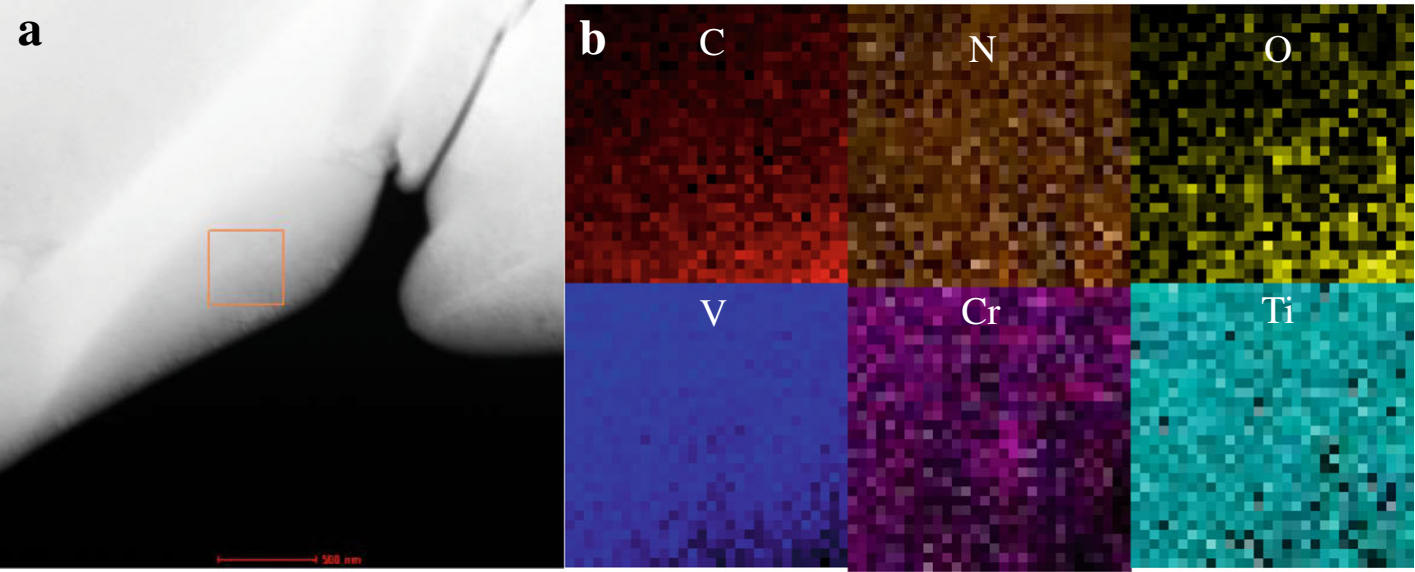
STEM and EDS mapping of V-4Cr-4Ti alloys after post-irradiation annealing treatment at 450 °C for 30 h. **a** The low-magnification Z-contrast image. **b** The composition mapping

From Fig. 5, no obvious precipitates appeared. Although the content of oxygen was a little high, there is no plate or disc-like precipitates. The quantitative analysis of irradiation-induced defects is as follows.

### Irradiation Hardening

**Fig. 6:**
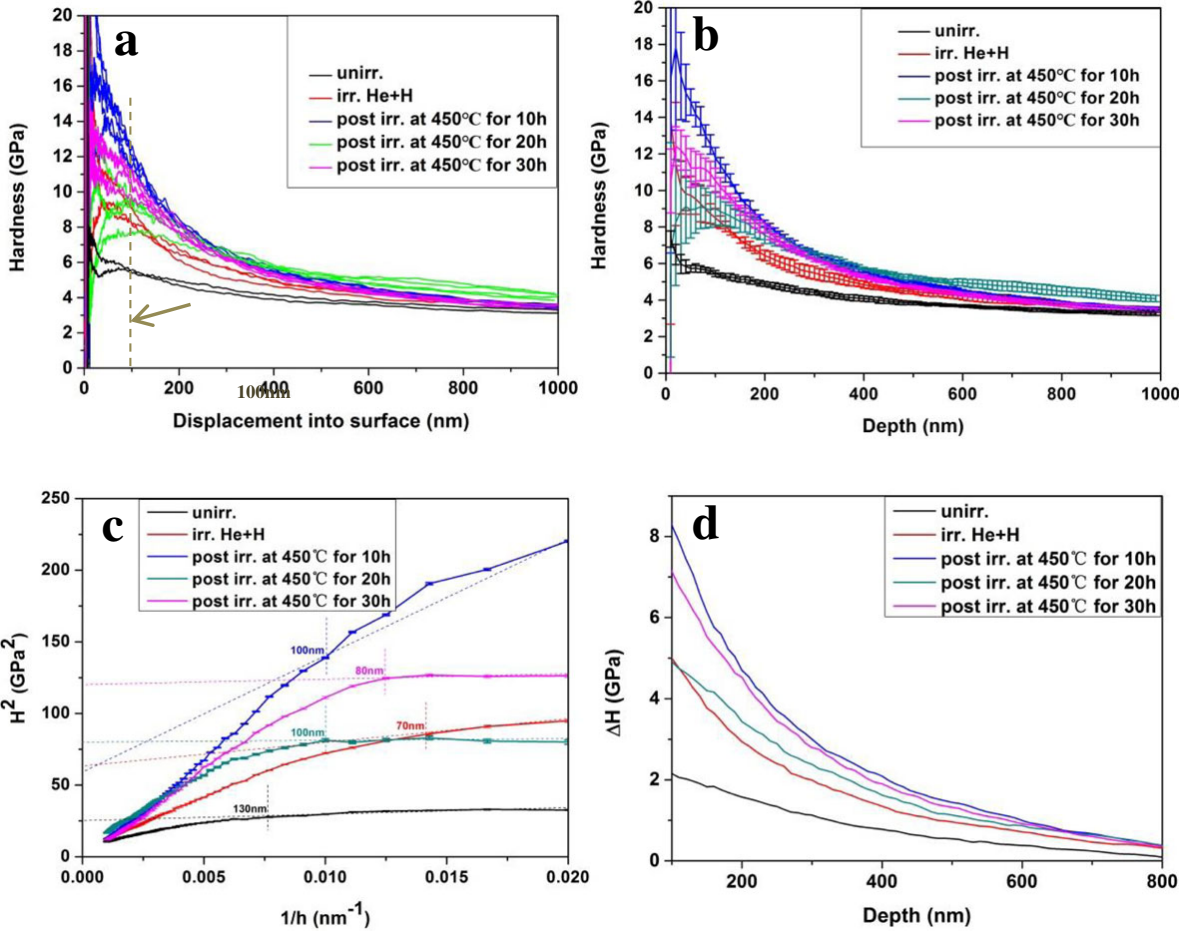
Hardness in V-4Cr-4Ti alloys with different conditions. **a** Depth profiles of raw irradiation hardness. **b** Indentation depth of average nanoindentation hardness with error bar. **c** Plot of *H*^2^ vs 1/h for irradiated specimens. **d** Experimental Δ*H* measurements corrected for ISE

Nanoindentation test was used to test the hardening of as-irradiated and post-irradiated samples in this study by reason of small irradiated area and limit irradiated depth of sample in ion accelerator. The results are shown in Fig. 6. For the sake of comparison, the hardness of un-irradiated V-4Cr-4Ti alloy was also tested.

Indentation size effect (ISE) was observed for almost all the samples from Fig. 6a, manifesting as the smaller indents gave higher reading in hardness. To exclude ISE, the data in region shallower than 100 nm was ignored. The depth dependent of average nanoindentation hardness with error bar for all the samples is given in Fig. 6b. It is obvious that the hardening was induced by irradiation. The hardness of as-irradiated and post-irradiated samples is higher than that of un-irradiated samples.

The results of hardness were further corrected using Nix-Gao model [25] which explained the increasing hardness due to the geometrically necessary dislocations near the surface that accommodate the indenter [26]. The Nix-Gao model is expressed as:

1 $$ {H}^2={H}_0^2\left(1+\frac{h^{\ast }}{h}\right) $$

Where *H* is the experimental hardness, *H*_0_ is the hardness at infinite depth, h^*^ is a characteristic length which depends on the material and the shape of indenter tips, and *h* is the indentation depth.

If *H*^2^ is set as *Y*-axis, while 1/h is set as *X*-axis, the plot of *H*^2^ vs 1/h for all samples was obtained as shown in Fig. 6c. It can be seen that the plot of *H*^2^ vs 1/h shows good linearity at shallower depth and deviates from linear fitting at deeper region [6, 27].

The degree of deviation in Fig. 6c for irradiated samples was larger. *H*_0_ in irradiated region can be obtained by fitting the corresponding data from Fig. 6c. Then, we can get experimental ΔH measurements corrected for ISE shown in Fig. 6d. The hardness of un-irradiated samples was the lowest, then as-irradiated sample, which indicated the increment of hardness, was induced by irradiation. Among the three series of samples with post-irradiation annealing, the hardness of samples after post-irradiation annealing at 450 °C for 20 h was lowest, and when the holding time was 10 h, the hardness was highest. The differences may be caused by the interaction among point defects, dislocation loops, and bubbles. We discussed it below through the dispersed barrier hardening model and Friedel-Kroupa-Hirsch relationship.

Dislocation loops and bubbles are contributed to irradiation hardening. So, we analyzed the irradiation hardening of numerical simulation from two aspects. On the basis of dispersed barrier hardening model, we can estimate the increasing of yield stress [28] caused by dislocation loops.

2 $$ \varDelta {\sigma}_y= M\alpha \mu b/1= M\alpha \mu b\sqrt{Nd} $$

Where, *M* is Tarlor factor (3.05 for BCC metal); *α* is the barrier strength (0.45), I is the average spacing between obstacles which can be estimated as 1/$\sqrt{Nd}$, *μ* is the shear modulus, *b* is Burgers vector, and *N* and *d* are the average loop density and mean size of dislocation loops, respectively, which are shown in Table 2. According to the formula, the hardening induced by dislocation loops is proportional to $\sqrt{Nd}$.

**Table 2:** Quantitative analysis of irradiation-induced defects

Holding time (h)	Dislocation loop		Bubble		Precipitation
	Size (nm)	Number density (nm^−2^)	Size (nm)	Number density (nm^−2^)	
0	*	*	×	×	×
10	4	5.2 × 10^−3^	9	1.5 × 10^− 3^	×
20	18	7.5 × 10^−4^	11	2.1 × 10^−3^	×
30	×		14	1.8 × 10^−3^	×

*Too small to distinguish

The hardening induced by bubbles can be developed by Friedel-Kroupa-Hirsch relationship.

3 $$ \Delta  \sigma =\frac{1}{8} M\mu bd{N}^{\frac{2}{3}} $$

where *N* and *d* are the average loop density and mean size of bubbles which are shown in Table 2.

According to formulas (2) and (3), the irradiation hardening of V-4Cr-4Ti alloy that underwent the post-irradiation annealing treatment for 10, 20, and 30 h at 450 °C was estimated, as follows. A and B represent different constants in formulas (2) and (3).

From Table 3, the influence of dislocation loops on irradiation hardening was reduced and the impact of bubble was the opposite with the holding time. It is noting that the calculation did not include the un-irradiated and as-irradiated alloy because we could not count up the size and number density of dislocation loops and bubbles in them.

**Table 3:** The evaluation of irradiation hardening of V-4Cr-4Ti alloy that underwent the post-irradiation annealing treatment for 10, 20, and 30 h at 450 °C

Holding time (h)	Dislocation loop	Bubble
10	4.6A	54.7B
20	3.7A	83.7B
30	×	96.2B

A and B represent different constants in formulas (2) and (3)

Without post-irradiation annealing, there were small defects or dislocation loops at incubation period. Lattice distortion caused by irradiation defects influenced the irradiation hardening. When annealed at 450 °C, dislocation loops grew up. And the bubbles emerged and coarsened. Bubble growth was through helium induced loop punching, aided by the presence of hydrogen, instead of as a direct interaction between hydrogen and helium [19]. Interaction between bubble and loops was strong when the holding time was 10 h and would increase the hardening. The continued holding time made vacancies and interstitials annihilated at all kinds of sinks such as loops, bubbles, grain boundary, and free surface. The defects left were less and less. Meanwhile, dislocation loops escaped from surface slowly. The pinning effect between dislocation loops and bubbles became weaker which caused the minor recovery of irradiation hardening. When the holding time was up to 30 h, most dislocation loops disappeared. Then, very large bubbles played a dominant role on hardening.

Although the hardening of irradiated V-4Cr-4Ti alloys is lower than that of irradiated China low activation martensitic steel [29], the irradiation hardening did not recover according to the annealing at 450 °C for up to 30 h. Fukumoto et al. [14] studied post-irradiation annealing treatment of neutron-irradiated vanadium alloys and found 3% elongation recovery in V-4Cr-4Ti alloys that was achieved by the annealing treatment at 500 °C for 20 h in a vacuum. However, microstructural elements (e.g., defect clusters and dislocation structures) retained high hardening even after 50 h of annealing treatment. Further research are needed to be conducted considering to increase the annealing temperature [11] or extend the holding time.

## Conclusions

V-4Cr-4Ti alloy was irradiated He and H ion irradiation sequentially to a dose of 10^17^ions/cm^2^ at RT and then carried out post-irradiation annealing at 450 °C for 10–30 h to evaluate the evolution of microstructure and hardening. Dislocation loops and bubbles formed in post-irradiated annealing V-4Cr-4Ti alloy. The size of dislocation loops and bubbles increased gradually with increasing holding time while the number density of dislocation loops and bubbles decreased. At last, large dislocation loops migrated to free surface. HRTEM observations showed that dislocation lines left in the matrix. Bubbles combined with each other and coarsened. Ion irradiation and post-irradiation annealing induced the evolution of hardening that was found by nanoindentation test. The irradiation hardening corresponded to the microstructural changes. Without post-irradiation annealing, lattice distortion induced by point defects caused irradiation hardening. As the annealing treatment at 450 °C proceeded for 10 h, the hardness increased because the pinning effect between dislocation loops and bubbles was strong. When the holding time was up to 20 h, the hardening recovered a little compared with 10 h annealing. At that moment, the interaction between dislocation loops and bubbles was weak. With an annealing time of 30 h, the hardening increased again and the influence of bubbles is dominant.

## Abbreviations

H: Hydrogen
He: Helium
He-V: He-vacancy
HRTEM: High-resolution transmission electron microscopy
ISE: Indentation size effect
RT: Room temperature
SRIM: Stopping and Range of Ions in Matter
STEM-EDS: Energy dispersive X-ray spectrum of scanning electron microscope
TEM: Transmission electron microscope
